# Three months of weekly rifapentine plus isoniazid for TB prevention among people with HIV

**DOI:** 10.5588/ijtldopen.23.0609

**Published:** 2024-09-01

**Authors:** R. Borse, B. Randive, S. Mattoo, P. Malik, H. Solanki, A. Gupta, R.E. Chaisson, V. Mave, N. Suryavanshi

**Affiliations:** ^1^ Byramjee Jeejeebhoy Government Medical College, Pune, India;; ^2^ Byramjee Jeejeebhoy Government Medical College-Johns Hopkins University Clinical Research Site, Pune, India;; ^3^ Center for Infectious Diseases India, Pune, India;; ^4^ Central TB Division, Ministry of Health & Family Welfare, Government of India, New Delhi, India;; ^5^ World Health Organization, Country Office for India, New Delhi, India;; ^6^ Johns Hopkins University, School of Medicine, Baltimore, MD, USA.

**Keywords:** 3HP, TB preventive therapy, TB prevention in PLHIV

## Abstract

**BACKGROUND:**

Evidence on implementation of three months of weekly isoniazid (H, INH) and rifapentine (P, RPT) (3HP) as a TB preventive therapy (TPT) for at-risk groups in Indian programmatic conditions is limited.

**METHODS:**

A prospective demonstration study assessing scale-up, safety, and effectiveness of 3HP TPT among people living with HIV (PLHIV) in Indian programmatic settings was conducted.

**RESULTS:**

Of 656 screened PLHIV, 502 (77%) received 3HP. Of these, 20 (4%) discontinued TPT due to toxicity,17 (3.8%) lost to follow-up, one (0.2%) had breakthrough rifampicin-sensitive TB, and 464 (92%) completed 3 HP TPT. Of 288 (57%) overall adverse events (AEs), 46 (9%) had Grade 2 or above AEs. The median time to AE was 14 days (IQR 7–42). Serious adverse events (SAEs) were reported in 9 (2%) participants; of these, 7 (78%) were not related to 3HP. No TB episodes occurred during the 1-year follow-up period.

**CONCLUSION:**

3HP TPT completion rate of 92%, with few adverse events leading to 3HP discontinuation, providing evidence of the scalability and safety of 3HP TPT among PLHIV in Indian health program settings.

## RÉSUMÉ

**CONTEXTE:**

Les données probantes sur la mise en œuvre de 3 mois de traitement hebdomadaire d'isoniazide (H, INH) et de rifapentine (P, RPT) (3HP) en tant que traitement préventif de la TB (TPT) pour les groupes à risque dans les conditions programmatiques indiennes sont limitées.

**MÉTHODES:**

Une étude de démonstration prospective évaluant la mise à l'échelle, l'innocuité et l'efficacité de la TPT 3HP chez les personnes vivant avec le VIH (PVVIH) dans des contextes programmatiques indiens a été menée.

**RÉSULTATS:**

Sur les 656 PVVIH dépistés, 502 (77%) ont reçu le traitement 3HP. Parmi eux, 20 (4%) ont interrompu le TPT en raison de toxicité, 17 (3,8%) ont été perdus de vue, un (0,2%) a développé une TB sensible à la rifampicine et 464 (92%) ont terminé le TPT 3HP. Parmi les 288 (57%) événements indésirables (AE, pour l’anglais, « adverse events ») en tout, 46 (9%) ont présenté des AE de Grade 2 ou supérieur. Le délai médian jusqu'à l'apparition d'un AE était de 14 jours (IQR 7–42). Des événements indésirables graves ont été rapportés chez 9 (2%) participants ; parmi eux, 7 (78%) n'étaient pas liés au traitement 3HP. Aucun épisode de TB n'a été observé pendant la période de suivi d'un an.

**CONCLUSION:**

Le taux d'achèvement du TPT 3HP est de 92%, avec peu d'événements indésirables conduisant à l'arrêt du TPT 3HP, fournissant des preuves de l'évolutivité et de l'innocuité du TPT 3HP chez les PVVIH dans le cadre de programmes de santé indiens.

TB remains among the leading causes of morbidity and mortality from an infectious disease pathogen worldwide. The Global Tuberculosis Report 2023 estimates that 10.6 million people fell ill with TB in 2022, of which 6% were people living with HIV (PLHIV).^1^ More than two-thirds of these people with TB were from eight countries, with India ranking first, accounting for 27% of the total global burden.^1^ Furthermore, India contributed to 48,000 TB cases of PLHIV and 11,000 HIV-associated TB deaths.^1^

TB preventive therapy (TPT) to treat TB infection (TBI)^2,3^ is a cornerstone for accelerating ending TB,^4^ specifically among PLHIV, who have a substantial risk for progression to TB disease.^5^ The WHO recommends TPT for all PLHIV^6,7^ after ruling out active disease.^8^ Isoniazid preventive treatment (IPT) for 6 months (6H) has been a widely used regimen under programmatic conditions, including in PLHIV; however, its effectiveness is limited by lower (<50%) treatment completion rates^9^ and challenges in implementation due to the 6-month duration of TPT.^10–13^ A shorter course regimen recommended by WHO, 3 months once-weekly rifapentine (P) and isoniazid (H) (3HP),^7^ is non-inferior to 9 months of IPT and is well tolerated with better adherence and completion rates.^14–17^ In 2021, India's National TB Elimination Programme (NTEP) included 3HP as an option among household contacts (HHC), PLHIV and other risk groups.^18^ Although studies have reported the safety, tolerability, and effectiveness of 3HP among PLHIV in clinical trial conditions, information on its implementation in Indian programmatic conditions is limited. To examine the scale-up, safety, and effectiveness of 3HP in programmatic settings, a demonstration project of providing short course TPT among PLHIV was initiated in one of the largest ART centres in India.

## METHODS

A prospective demonstration study was conducted between December 2021 and July 2023 at the antiretroviral therapy (ART) centre of a public tertiary care hospital, which cares for about 6,200 PLHIV residing in the Pune District of Maharashtra State. Participants aged >2 years were included in the study if they had laboratory confirmation of HIV, attended the ART centre, had no current evidence of active TB, were not currently or previously on IPT, and were willing to participate in study procedures by providing written informed consent. We excluded those who were critically ill, had a body weight <10 kg, were a household contact of known MDR-TB/rifampicin or isoniazid-resistant TB, had a history of seizure disorder or peripheral neuropathy (Grade 3 or 4), those on nevirapine and or protease inhibitor-based ART regimen, or any medication known to interact with rifapentine. Pregnant and breastfeeding mothers and those consuming regular alcohol were also excluded. In addition, those with baseline investigation showing haemoglobin <7.5 g/dL, alanine aminotransferase (ALT) >2 times the upper limit of normal (ULN), total/direct bilirubin >2 times the ULN, platelet count <100,000/mm^3^, blood urea and or serum creatinine >ULN, were excluded.

Upon enrolment, participants received the first dose of 3 months of once-weekly isoniazid and rifapentine (3HP) under the observation of clinic staff, and the remaining three weekly dosages for that month were self-administered at home with study staff performing weekly telephonic reminders. The doses were administered along with ART medicines. After that, doses were dispensed monthly for the remaining 2 months. Participants were monitored for any adverse drug reactions or the onset of TB symptoms every month during the 3HP treatment at the time of their visit to the ART centre for ART and at Months 6 and 12 post-3HP initiation. Three pills of a fixed-dose combination (FDC) of HP (300 mg/300 mg per pill) along with pyridoxine were used for participants >14 years of age. Participants were asked to contact the study clinician immediately if they developed any symptoms or illness after consuming 3HP. All serious adverse events were documented and reported to the institutional ethics committee within twenty-four hours of the site notice. We used The Division of AIDS (DAIDS) Table for Grading the Severity of Adult and Pediatric Adverse Events, Version 2.1, as a reference for grading AE severity.^19^ TB incidence was documented for 1 year from the first 3HP dose. The study was approved by the institutional ethics committees and national regulatory authorities. Written informed consent was obtained from all study participants.

### Statistical analysis

Analysis in this manuscript is restricted to the PLHIV population who were 18 years of age or older at the time of study participation. A descriptive analysis was done to present the study population's demographic characteristics and the safety, completion rate, and effectiveness of the 3HP. Safety analysis was performed on all participants who received ≥1 dose of 3HP. The 3HP completion rate was estimated as the proportion of participants completing at least eleven 3HP doses within 16 weeks after the first 3HP dose. Safety was estimated as the proportion of participants discontinued due to drug toxicity among those who received at least one dose of 3HP. TB incidence among those who completed at least 11 doses of 3HP is used to assess the 3HP effectiveness over a 1-year follow-up. Student's *t-*test and the Pearson χ^2^ tests were used to evaluate the difference between the PLHIV group who completed and did not complete the 3HP treatment. Logistic regression analysis was conducted to examine the factors associated with 3HP discontinuation. Statistical analyses were performed using Stata version 15.1

## RESULTS

A total of 656 PLHIVs were screened, and 113 (17%) were not started on 3HP as they were ineligible due to abnormal clinical conditions, mainly blood urea and or serum creatinine >ULN (6%), ALT >2 times ULN (2%), platelet count <100,000/mm^3^ (2%), haemoglobin <7.5 g/dL (2%), comorbidities or being on medications known to interact with rifapentine (2%), and new evidence of active TB (1%) (Figure). Forty-one (6%) participants were not started on 3HP; either they declined to start 3HP or did not visit the ART centre within a window period of one month after the screening. The 3HP was prescribed to 502 (77%) participants, and their characteristics are shown in Table 1. Of these, 499 (99%) were on stable ART, and 490 (98%) were on dolutegravir-based regimens. The median age of participants was 41.5 years (interquartile range [IQR] 33–49), the median CD4 count was 492 cells/mm^3^ (IQR 291–699), and 352 (70%) had undetectable viral load at study entry.

**Figure.**
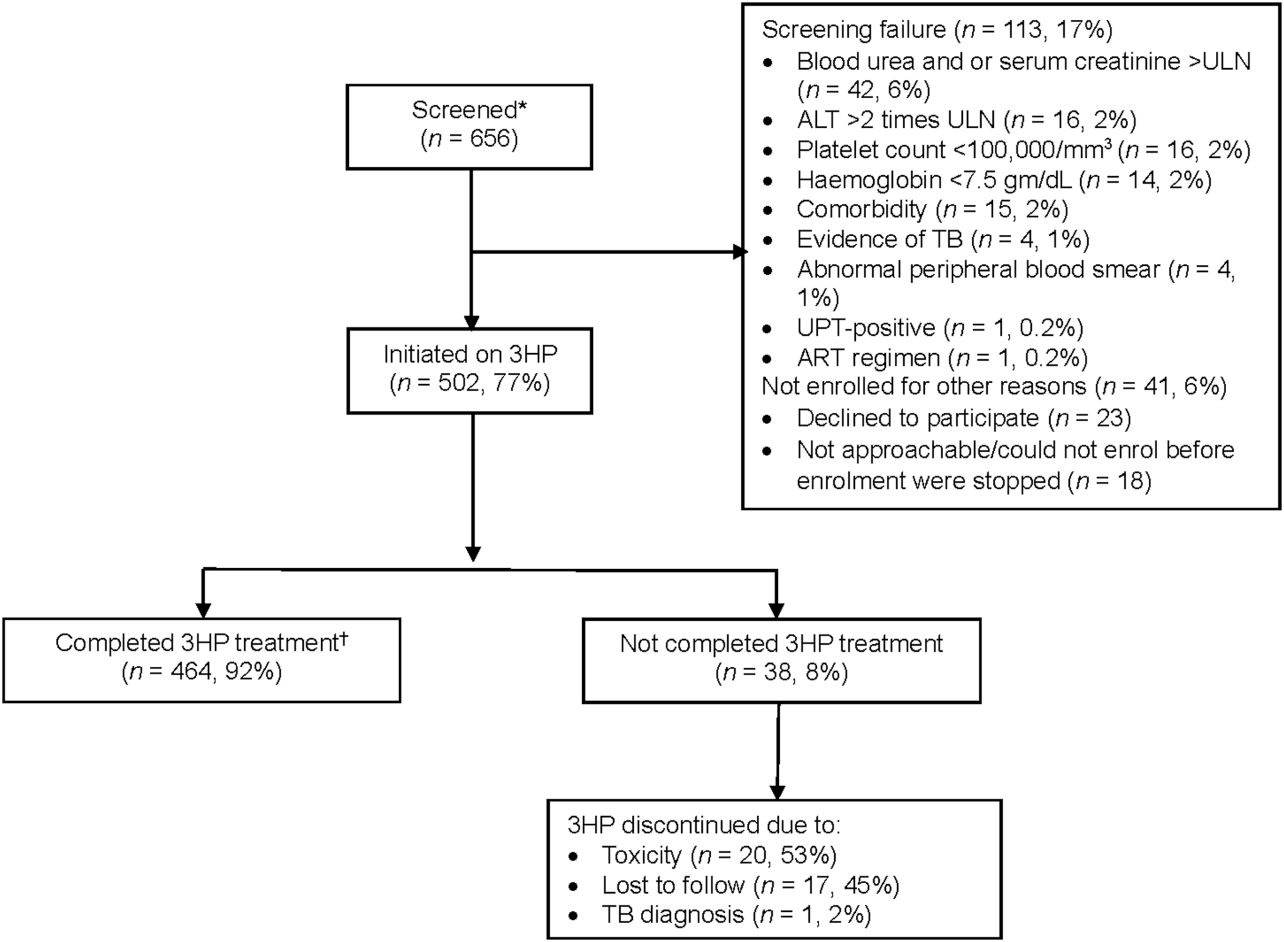
Study flow diagram. *PLHIV who consented to participate and were evaluated for eligibility. ^†^Included those who have completed at least 11 doses of 3HP within 16 weeks from the first dose. ULN = upper limit of normal; ALT = alanine aminotransferase; UPT = urine pregnancy test; ART = antiretroviral therapy; 3HP = 3 months of isoniazid and rifapentine; PLHIV = people living with HIV.

**Table 1.** Demographic and clinical characteristics of adult PLHIV participants who completed and those who did not complete the 3HP TPT course (*n* = 502).*

Characteristics	Initiated on 3HP	Completed^†^ 3HP	Not completed 3HP^‡^	Unadjusted OR (95% CI)	Adjusted OR (95% CI)
	(*n* = 502)	(*n* = 464)	(*n* = 38)		
	*n* (%)	*n* (%)	*n* (%)		
Age, years, mean (95% CI)	41 (40–42)	41 (40–42)	43 (39–47)	1.01 (0.99–1.04)	1.00 (0.97–1.04)
Weight, kg, mean (95% CI)	55 (54–56)	55 (54–56)	53 (49–57)	0.98 (0.95–1.01)	0.98 (0.94–1.01)
Sex					
Male	189 (38)	172 (91)	17 (9)	1	1
Female	313 (62)	292 (93)	21 (7)	0.73 (0.37–1.41)	0.63 (0.26–1.47)
Comorbidities					
Diabetes mellitus					
No	497 (99)	461 (93)	36 (7)^§^	1^§^	1^§^
Yes	5 (1)	3 (60)	2 (40)^§^	8.5 (1.38–52.74)^§^	12.82 (1.22–134.75)^§^
History of TB treatment					
No	358 (71)	334 (93)	24 (7)	1	1
Yes	144 (29)	130 (90)	14 (10)	1.49 (0.75–2.98)	1.94 (0.89–4.23)
HIV characteristics					
CD4 count, cells/mm^3^					
<200	63 (13)	54 (85.7)	9 (14.3)	1	1
200–<350	93 (18)	86 (92.5)	7 (7.5)	0.49 (0.18–1.39)	0.41 (0.13–1.29)
>350	346 (69)	324 (93.6)	22 (6.36)	0.41 (0.18-0.93)^§^	0.39 (0.14-1.09)
Baseline viral load					
Not detectable	352 (70)	332 (94.3)	20 (5.7) ^§^	1^§^	1
Detectable	150 (30)	132 (88.0)	18 (12.0) ^§^	2.3 (1.16-4.41)^§^	2.39 (1.02-5.63)^§^
Adverse events					
No	214 (43)	204 (95.3)	10 (4.7)^§^	1	1
Grade I	242 (48)	229 (94.6)	13 (5.4)^§^	1.16 (0.49–2.69)	1.39 (0.57–3.44)
Grade II and above	46 (9)	31 (67.4)	15 (32.6)^§^	9.87 (4.07-23.91)^§^	15.35 (5.59-42.17)^§^
ART regimen at 3HP initiation					
Abacavir (ABC)	11 (2)	10 (90.9)	1 (10.1)		
Tenofovir+ lamivudine+ dolutegravir (TLD)	464 (92)	430 (92.7)	34 (7.3)		
Tenofovir+ lamivudine+ efavirenz (TLE)	1 (0.2)	1 (100)	0 (0)		
Zidovudine+ lamivudine + dolutegravir (ZLD)	26 (5)	23 (88.5)	3 (11.5)		

* Logistic regression was performed to assess the relationship between participant characteristics and 3HP TPT discontinuation. Participants (*n* = 502) who were started on 3HP TPT were included in the analysis.

† Includes those who had completed at least 11 doses of 3HP within 16 weeks from the first dose.

‡ Includes those who could not complete 11 doses within 16 weeks due to various reasons.

§ Statistically significant.

PLHIV = people living with HIV; 3HP = 3 months of isoniazid and rifapentine; TPT = TB preventive therapy; OR = odds ratio; CI = confidence interval; ART = antiretroviral therapy.

Minor (Grade 1) AEs were seen in 242 (48%) of participants, most commonly gastrointestinal (15.1%), hypersensitivity (itching/skin rashes) (11.7%), flu-like reaction (6.8%), and peripheral neuropathy (5.2%); of these, only four discontinued 3HP TPT due to AEs. Moderate (Grade 2 AEs or above) were seen among 46 (9%) participants and included gastrointestinal (3.2%), hypersensitivity (itching/skin rashes) (1.4%), peripheral neuropathy (1.2%), flu-like reaction (0.4%), hepatotoxicity (0.4%); of these 15 discontinued 3HP due to AEs related to 3HP. The median time for Grade 2 and above events was 14 days (IQR7–42). Serious adverse events (SAEs) requiring hospitalisation and one death were reported in nine (1.8%) participants; to note, seven (78%) of these SAEs were not related to 3HP, two (22%) related SAEs were hospitalised for nausea and vomiting leading to discontinuation of 3HP (Table 2). The participant who died during 3HP treatment did not reveal alcoholic status at screening; however, later hospitalised for alcoholic hepatitis, leading to immediate discontinuation of 3HP. The participant was discharged against medical advice twice and continued consuming alcohol, leading to death at home more than a month after 3HP discontinuation (Table 2).

**Table 2.** SAEs reported among participants receiving 3HP.

SAE	Age Years	Sex	CD4 count (cells/mm^3^)	Viral load Copies/ml	Time to event Days	Whether related to 3HP study drugs?*	Outcome
Hospitalisation to investigate the reason for amenorrhoea	45	F	212	Undetectable	30	No	Diagnosed as perimenopausal amenorrhoea
Hospitalisation for nausea and vomiting	58	F	637	Undetectable	14	Yes	Resolved
Hospitalisation for flank pain due to trauma	41	F	559	Undetectable	56	No	Resolved
Hospitalisation for MTP	26	F	692	Undetectable	96	No	Resolved; MTP done
Hospitalisation for uterine prolapse	43	F	470	1956	157	No	Resolved: hysterectomy done
Hospitalisation for liver cirrhosis	61	M	441	NA	47	No	Discharged against medical advice twice and died at home
Hospitalisation for perianal abscess with fistula	61	M	355	150	244	No	Resolved
Hospitalised for haemorrhoidectomy	47	F	646	Undetectable	80	No	Resolved
Hospitalisation for gastritis	48	F	549	Undetectable	51	Yes	Resolved

* Any hospitalisation/death during 3HP treatment was reported as SAE irrespective of its relationship with 3HP treatment.

SAE = serious adverse events; F = female; M = male; MTP = medical termination of pregnancy.

Of the 502 participants who started on 3HP, 20 (4%) participants discontinued 3HP because of 3HP drug toxicity, including gastrointestinal (1%), peripheral neuropathy (0.8%), hypersensitivity (0.6%), hepatotoxicity (0.4%), flu-like reaction (0.2%) and change in participant's ART to a regime contraindicated with 3HP (0.2%). The median time to discontinuation due to toxicity was 22 days (IQR 14–42), and 40% (8/20 individuals discontinued within three doses of 3HP. The 3HP was observed safe in the remaining 96% (482/502) participants. In addition to discontinuation due to toxicity, one participant (0.2%) discontinued due to breakthrough rifampicin-susceptible TB, i.e. TB diagnosis during TPT before treatment completion, and 17 (3.5%) were lost to follow-up. A total of 38 (8%) participants did not complete the 3HP schedule either due to drug toxicity or other reasons (Table 1). Thus, the treatment completion rate was 92% (464/502). The 3HP discontinuation in PLHIVs with detectable viral load was significantly higher than those with non-detectable (12.0% vs 5.7%; *P* = 0.01). Participants with Grade II or above AEs have a higher proportion of discontinuation than those with Grade I or no AEs (32.6% vs 5.4% or 4.7%; *P* = 0.00). The Grade II and above AE (OR 15.3, 95% confidence interval [CI] 5.59–42.2), detectable levels of viral load (OR 2.4, 95% CI 1.02–5.63), and diabetes (OR 12.8, 95% CI 1.2–134.7) were significantly associated with 3HP discontinuation (Table 1). No TB episodes occurred during the 1-year follow-up period post-3HP initiation.

## DISCUSSION

TB remains the most significant cause of morbidity and mortality among PLHIV residing in low- and middle-income settings such as India. Multiple clinical trials of TPT with 3HP have demonstrated efficacy, safety, and high completion rates compared to 6–9 months of daily isoniazid prophylaxis.^13,14,20,21^ None of these trials, however, included participants from India, the country with the world's highest TB burden and third largest burden of PLHIV, with an estimated 2.5 million PLHIV and 66,000 new HIV infections occurring annually. This demonstration study showed that a fixed-dose combination of 3HP can be safely implemented with high adherence and completion rates (92%) among PLHIV. To our knowledge, this study is the first to provide evidence about the use of 3HP TPT in Indian healthcare settings, providing critical evidence to inform the nationwide policy for scale-up of 3HP TPT among PLHIV.

An imperative consideration while scaling up any drug intervention in the public health program is to demonstrate comparable outcomes between real-world scenarios and clinical trial conditions. Comparing findings from prior clinical trials^20^ and implementation studies, we observed a 92% completion rate of 3HP and a lower rate of 3HP discontinuation due to toxicity (4%) when this TPT regimen was largely self-administered among PLHIV. Self-administered doses were utilised in this pragmatic study to simulate a real-world scenario and are backed by evidence that comparable TPT completion rate and drug toxicity profiles were observed between self-administration and directly observed doses.^22^

Even though a large number of participants experienced adverse events, the majority were Grade 1 events that did not require discontinuation of 3HP TPT, and similar observations have been reported by another 3HP evaluation study.^23,24^ Fewer than 10% experienced Grade 2 or above events, and even fewer participants (3.8%) discontinued 3HP due to AEs, signifying that this TPT regimen is safe to provide among PLHIV.^20,25,26^ To note, SAEs occurred in very few participants, of which only two cases were related to 3HP TPT. Our data are very similar to what has been observed in other studies.^27^ Furthermore, almost all participants were on dolutegravir-based regimen when 3HP TPT was concurrently administered, and only one participant had an ART regimen change due to an unrelated event, providing robust evidence for 3HP TPT for PLHIV who are on dolutegravir-based regimen. Previous pharmacokinetics studies of 3HP among PLHIV recommend that 3HP can be given to PLHIV taking dolutegravir-based antiretroviral therapy without dose adjustments.^28^

A notable finding of our study is that about 17% of screened participants were not started on 3HP due to abnormal baseline clinical assessments, including elevated liver enzymes observed in 2%. Although 3HP is less hepatotoxic than 9 months of isoniazid TPT, elevated baseline aspartate aminotransferase (AST) and ALT are known risk factors for hepatotoxicity. It, therefore, is prudent to perform baseline liver function tests (LFTs) among PLHIV before initiating 3HP TPT, in addition to ruling out active TB.^29^ Indeed, current Indian NTEP guidelines for TPT strongly encourage baseline LFTs for individuals having risk factors, including a history of liver disease, regular use of alcohol, chronic liver disease, or HIV infection,^18^ and this should be continued when the 3HP TPT regimen gets rolled out in India.

Our study has some limitations. Although cost-related data was not documented in our study, previous cost-effectiveness modelling comparing four regimens for TPT found that self-administered, once-weekly 3HP had an economic advantage over other regimens if adherence remained high and toxicity did not increase, as observed in this study.^30–32^ Furthermore, there was no comparison group in our study as we aimed to demonstrate the implementation feasibility of scaling up shorter TPT regimen intervention in programmatic settings rather than comparing with other TPT regimens. Similar studies assessing the implementation of currently available 6H TPT for PLHIV reported lower uptake of TPT ranging between 40%-76%, but a comparable completion rate of 81–89%.^33–35^ The duration of follow-up was 1 year, which may have limited the assessment of measuring TB incidence and resulting drug resistance amplification, if any.

In conclusion, our study among PLHIV demonstrated that self-administered 3HP is safe, feasible, and has a more than 90% completion rate with a negligible failure rate. Careful consideration should be given to assessing baseline liver enzymes before 3HP TPT initiation and regular monitoring for adverse events among PLHIV. Importantly, our study provides timely insights about the feasibility of 3HP rollout among PLHIV on dolutegravir-based ART and further supports the NTEP efforts to scale up 3HP TPT more widely in India.
